# Advances in Platelet Subpopulation Research

**DOI:** 10.3389/fcvm.2019.00138

**Published:** 2019-09-13

**Authors:** Gabriela Lesyk, Paul Jurasz

**Affiliations:** ^1^Faculty of Pharmacy and Pharmaceutical Sciences, University of Alberta, Edmonton, AB, Canada; ^2^Cardiovascular Research Centre, University of Alberta, Edmonton, AB, Canada; ^3^Department of Pharmacology, Faculty of Medicine and Dentistry, University of Alberta, Edmonton, AB, Canada; ^4^Mazankowski Alberta Heart Institute, University of Alberta, Edmonton, AB, Canada

**Keywords:** platelet subpopulations, vanguard and follower platelets, COAT-platelets, eNOS-based platelet subpopulations, thrombosis, hemostasis

## Abstract

Although lacking a nucleus, platelets are increasingly recognized not only for their complexity, but also for their diversity. Some 50 years ago platelet subpopulations were characterized by size and density, and these characteristics were thought to reflect platelet aging. Since, our knowledge of platelet heterogeneity has grown to recognize that differences in platelet biochemistry and function exist. This includes the identification of vanguard and follower platelets, platelets with differing procoagulant ability including “COAT-platelets” which enhance procoagulant protein retention on their surface, and most recently, the identification of platelet subpopulations with a differential ability to generate and respond to nitric oxide. Hence, in this mini-review, we summarize the current knowledge of platelet subpopulation diversity focusing on their physical, biochemical, and functional heterogeneity. In addition, we review how platelet subpopulations may change between health and disease and how differences among platelets may influence response to anti-platelet therapy. Finally, we look forward and discuss some of the future directions and challenges for this growing field of platelet research.

## Introduction

Compared to leukocytes, which exist as functionally distinct subpopulations, platelets have often been considered simple. This general apathy for platelet complexity may stem from the fact that platelets lack nuclei and because they are so widely recognized for their roles in hemostasis and thrombosis. However, increasingly platelets are recognized for their biochemical and functional complexity despite being anucleate. Platelet proteome and transcriptome studies have revealed that platelets contain nearly 4,000 different proteins (1, 2) and an abundance of mRNA (3, 4) and miRNA (5–7). It's also appreciated within the field that platelets play diverse roles beyond hemostasis including contributing to wound healing, angiogenesis, and immunity and inflammation (8–11). Pathophysiologically, they are also recognized to contribute to cancer metastasis (12, 13), as well as to various inflammatory disorders (14, 15). To fulfill these diverse roles the question arises whether there is predetermined biochemical diversity and functional heterogeneity among platelets? That is, do platelets subpopulations with predetermined specialized functions exist?

## Historical Perspectives

Initial studies into platelet subpopulations occurred 50 years ago and focused on characterizing human platelet subpopulations based on size and buoyant density and largely isolated these subpopulations by density gradient centrifugation. Studies by Karpatkin (16) showed that large, dense platelets have greater glycogen, orthophosphate and adenine nucleotide content than smaller less dense platelets. The biochemical processes of glycolysis, glycogenolysis, and protein synthesis were reported to be greater in large-dense platelets but no differences were observed in lipid content or synthesis. Large-dense platelets were shown to aggregate more than small-light platelets largely due to greater ADP release and lower ADPase activity (17). Subsequently, large-dense platelets were shown to adhere to collagen at a faster rate and this was thought to reflect more surface GPIa/IIa (integrin α_2_β_1_) on this subpopulation (18). Electron microscopy examination of light platelets demonstrated lower granule content than that of dense platelets, but not fewer mitochondria (19). Although, currently it's recognized that an anuclear programmed cell death limits platelet lifespan (20, 21), the biochemical, ultrastructural, and functional differences among platelets with differing size and density were thought to reflect platelet aging with large-heavy platelets representing young platelets recently released into the circulation while light-small platelets represented older platelets that have circulated for a number of days. Using rabbit platelets, Rand et al. (22) demonstrated that the least dense platelets contained less sialic acid than the densest. They suggested loss of surface sialic acid from the least dense platelets was a mechanism by which old platelets are recognized and removed from the circulation.

Studies by Penington et al. (23, 24) argued that in fact megakaryocycte heterogeneity was responsible for platelet heterogeneity and that platelet density does not change with aging. Based on morphometric studies, they contended that the three-ploidy classes of megakaryocytes (8n, 16n, 32n) differ in their organelle content concentration and relate to the density of their platelet progeny. Large-dense platelets would be expected to arise from 8n megakaryocytes with greater granule content, while small-light platelets arise from 32n megakaryocytes. This view was more in line with that of Paulus that thrombopoiesis is likely responsible for platelet heterogeneity and not aging in circulation, but that only a single platelet population exists and the only size heterogeneity is that inherent to the log normally distributed population (25). Consistent with this thesis, other studies demonstrated that in the steady state platelet density does not change with circulatory age (26, 27), while others still showed that platelet density may increase with time due to accumulation of 5-hydroxytryptamine (5-HT) (28).

In addition to categorizing platelets into subpopulations based on size and density, platelets were also separated into subpopulations based on volume using counterflow centrifugation. Platelets with larger volume were shown to have more rapid and complete aggregation, and to be slightly denser (29). These studies argued that fundamentally platelets of different volume have similar function but the absolute abilities to aggregate and secrete granular contents correlates with their volume (30). Further, it was argued that platelet size heterogeneity likely results from their production from megakaryocytes and that this influences platelet function independently of time within circulation (30, 31).

Several groups attempted to unify the hypotheses that both megakaryocyte heterogeneity and platelet time in circulation may contribute to platelet heterogeneity and the formation of size and density-based subpopulations (32–34). However, it's also important to note that some discrepancies in characterizing size and density-based platelet subpopulations may have occurred due to species differences. It appears rabbit platelets decrease in density with circulatory age, while in humans high-density platelets may be enriched with those that may have circulated longer and accumulated more 5-HT (28). Other characterization discrepancies may have also occurred due to methodology, and theoretical aspects of density separation need to be considered when interpreting early platelet subpopulation studies. As explained by Martin and Trowbridge if centrifugation is stopped before equilibrium is reached platelets may be separated based on a mixture of volume and density variation (35). This problem appears to be greater for discontinuous vs. continuous gradients as a greater time is needed to reach equilibrium necessary for separation of density-based subpopulations. Further, it has been suggested that platelets held at equilibrium between the opposing forces of the gravitational field and medium buoyancy may cause trauma to the platelet potentially causing secretion of granular contents; thus, potentially changing the characteristics of the isolated subpopulations (35).

## Characterization of Platelet Subpopulations Based on Differential Function and Biochemistry

In addition to being characterized by size and density, platelet subpopulations have also been characterized based on function. Using a biotinylation technique to label dog platelets *in vivo*, it was demonstrated that reactivity to thrombin declines with platelet circulatory age (36). Similarly, utilizing ^35^S-labeled rabbit platelets Hirsh et al. (37) demonstrated that younger platelets with lower ^35^S-specific activity adhered to collagen fibers more readily than older platelets. Alike, two human platelet subpopulations were shown to have different collagen adhering kinetics, with 20% of platelets adhering within 1 min and with a second larger subpopulation accounting for 80% of platelets adhering more slowly between 1 and 60 min (38). The biochemical basis of this finding was unknown; however, several explanations were proposed. The one favored was the existence of functionally discrete subpopulations due to the sharp discontinuity between the two adhesion phases and the simple kinetic behavior of the second phase. Using time-lapse videomicroscopy and a microchamber model, Patel et al. (39) defined platelets that first adhered and spread on collagen as “vanguard” platelets and those that subsequently tether to and spread on vanguard platelets or nearby collagen as “followers.” Whether vanguard and follower platelets represent functionally and biochemically distinct platelets was not investigated, but analysis of platelet deposition showed that adhesion events occurred randomly.

In addition to characterizing based on function, some studies characterized platelet subpopulations based on biochemical differences including the ability to bind adenosine and release 5-HT (40), as well as the presence or absence of acid phosphatase although no functional differences were observed (41). However, biochemical comparison of low vs. high density human platelets by Opper et al. (42) revealed that low density platelets have an enhanced intracellular Ca^2+^ response to thrombin, increased ADP-ribosylation of the inhibitory G-protein (G_iα1−3_) and rho A, and decreased ADP-ribosylation of the stimulatory G-protein (G_sα_), as well as lower levels of the phosphorylated form of vasodilator-stimulated protein compared to high density platelets. These biochemical differences likely further explain the authors' findings that low density platelets demonstrate enhanced aggregation in response to thrombin, weaker inhibitory effects and a smaller rise in cGMP to the nitric oxide (NO) donor sodium nitroprusside, and a smaller increase in cAMP in response to prostaglandin E1 compared to high density platelets (43, 44). The authors proposed that the biochemical basis of the functional heterogeneity between low and high density platelets depends on differences in their G- and phospho-protein signaling within stimulatory and inhibitory signaling pathways (42). Others suggested that the increased reactivity of low density platelets may be attributed in part due to elevated α-granule content (45), although another study suggested α-granule content is greater in high density platelets (46).

Similar to the findings of Opper et al. (42), which suggested differences in NO-signaling between low and high density platelet subpopulations, recently we identified human platelet subpopulations based on the presence or absence of endothelial nitric oxide synthase (eNOS-positive and -negative platelets) and the differential ability to produce NO (47). We showed that eNOS-negative platelets fail to produce NO, have a down-regulated soluble guanylate cyclase-protein kinase G-vasodilator-stimulated protein (sGC-PKG-VASP) signaling pathway, primarily initiate adhesion to collagen, more readily activate integrin α_IIb_β_3_, and secrete more of the platelet activating protease matrix metalloproteinase-2 (MMP-2) than their eNOS-positive counterparts. eNOS-positive platelets have an intact eNOS-sGC-PKG-VASP signaling pathway, are more abundant (~80% of total platelets) and form the bulk of an aggregate via greater cyclooxygenase-1-mediated signaling. However, eNOS-positive platelets ultimately limit aggregate size via NO generation. It appears that eNOS-based platelet subpopulations are of the same circulatory age as the levels of activated caspase-3, the downstream effector of the platelet internal apoptotic clock (21), within them are equal. Moreover, eNOS-negative and –positive platelet volume was found to be equal also (47).

**Figure 1:**
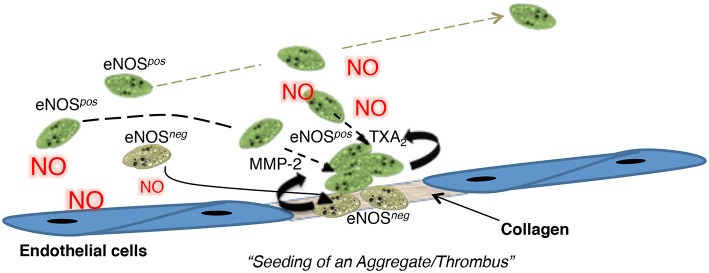
A cartoon summarizing the “seed platelet” hypothesis in which NO-refractory eNOS^*neg*^ platelets preferentially initiate platelet adhesion and aggregation, while eNOS^*pos*^ platelets form the bulk of an aggregate and limit its size. Figure S21. Radziwon-Balicka et al. (47), by permission of Oxford University Press. MMP-2, matrix metalloproteinase-2; NO, nitric oxide, TXA_2_, Thromboxane A_2_.

Based on these data, we proposed a novel model of thrombosis and hemostasis called the seed platelet hypothesis, in which eNOS-negative platelets initiate adhesion and aggregation reactions (Figure 1) (47). This initial response arises from their enhanced adhesiveness and reactivity due to an absence of endogenous NO generation (48, 49). Decreased sGC-PKG signaling within eNOS-negative platelets also facilitates refractoriness to endothelial-derived NO and increases integrin α_IIb_β_3_ activation (50, 51), which stabilizes initial rolling and adhesion (52). We further proposed that enhanced MMP-2 secretion by eNOS-negative platelets promotes eNOS-negative platelet activation and recruitment of eNOS-positive platelets to the forming aggregate (53–55). eNOS-positive platelets then form the bulk of an aggregate/thrombus due to their higher COX-1 content and greater thromboxane A_2_ generation. However, eNOS-positive platelets ultimately limit aggregate/thrombus size via NO generation as both increasing the ratio of eNOS^*neg*^ to eNOS^*pos*^ platelets and pharmacologically inhibiting eNOS enhances aggregation.

The differential ability of platelets to generate NO has been shown by others (56). However, how eNOS-based platelet subpopulations and the seed-platelet model of thrombosis and hemostasis relate to other models, such as a core and shell model of hemostatic plug formation by Stalker et al. (57), still needs to be determined. Similarly, what role these subpopulations play in coagulation and how they relate to subpopulations with differential procoagulant ability also remains to be seen (58).

## Characterization of Platelet Subpopulations Based on Differential Procoagulant Ability

For a thorough review of procoagulant platelets we refer readers to a paper by Reddy and Rand within this series of articles on the Established and Novel Roles of Platelets in Health and Disease. However, briefly, it has been long recognized that a subpopulation of platelets becomes procoagulant exposing a phosphatidylserine-(PS)-surface upon activation (59). Alberio et al. (60) described a platelet subpopulation demonstrating α-granule-derived surface-bound factor V (FV), which they named COAT-platelets, due to their appearance upon collagen and thrombin stimulation. This subpopulation accounts for approximately 30% of platelets and was shown to utilize 5-HT to retain other α-granule-derived procoagulant proteins such fibrinogen and thrombospondin on the platelet surface (58, 61). COAT-platelets may also retain factors VIII, IX, and X (62), and they may be generated via stimulation of platelets with thrombin alone (63). These platelets are also sometimes referred to as “coat” or “coated” platelets due to their surface retention of procoagulant proteins. Additionally, heterogeneity within the procoagulant subpopulation itself may exist wherein procoagulant platelets that are highly PS-positive and have a sustained increase in Ca^2+^-signaling surprisingly aggregate poorly, due in part to a lack of active integrin α_IIb_β_3_. Conversely, a COAT-platelet subpopulation with lower intracellular Ca^2+^-signaling exhibits greater proaggregatory potential (64). Using mathematical modeling to support their experimental observations, Yakimenko Alena et al. (65) and Abaeva et al. (66) suggested that highly PS-positive COAT-platelets are recruited into developing aggregates by non-COAT platelets, as a result of a high surface density of α-granule-derived fibrinogen/fibrin retained on the COAT-platelet surface, and largely do not bind each other. Others have demonstrated that in fact integrin α_IIb_β_3_ may initially activate but subsequently deactivate, while PS surface exposure occurs more slowly in these COAT-platelets (67). Nonetheless, a model of platelet-based coagulation was proposed by Heemskerk et al. (68) wherein two different subpopulations of platelets with differential roles exist (procoagulant vs. aggregating platelets). Collagen-adhered platelets, and later in the growing thrombus thrombin-activated platelets, expose PS on their membranes serving as a substrate for coagulation factors, thrombin generation, and fibrin coat formation. Aggregating platelets, due to their activated integrin α_IIb_β_3_, on the other hand are proposed to be responsible for contracting and retracting the clot by interacting with fibrin.

Whether all platelets have the capacity to become procoagulant/COAT vs. aggregating platelet subpopulations or whether these subpopulations are predetermined requires further investigation. Studying platelet adhesion to glass some have argued that all platelets can form these subpopulations and that they simply reflect snapshots in time of a dynamic platelet activation process (69). However, multi-parameter flow cytometry studies of platelet responses to increasing concentrations of thrombin and CRP-XL showed that only a fraction of platelets can take on the procoagulant phenotype supporting the theory of distinct platelet subpopulations (70).

## Changes in Platelet Subpopulations Between Health and Disease

Relatively little is known about how platelet subpopulations change between physiological and pathological conditions, although recently a number of studies have investigated procoagulant/COAT“ed” platelet levels in stroke and transient ischemic attack (71–78). Coated-platelets were demonstrated to be elevated in patients with large-artery atherosclerotic stroke compared to small artery lacunar strokes with the authors suggesting this reflects distinct pathological processes of ischemic stroke subtypes (71). Similarly, elevated coated-platelet levels were reported during transient ischemic attacks (TIA) (79). High coated-platelet levels were also reported to be associated with early stroke recurrence in large-artery stroke patients (72, 74); and in patients with asymptomatic carotid artery stenosis high coat-platelet levels (≥45% of platelets) improved stroke and TIA prediction (75). Conversely, a pilot study reported that non-lacunar stroke patients with early haemorrhagic transformation exhibited lower coated-platelet levels (78). Likewise, among subarachnoid hemorrhage and spontaneous intracerebral hemorrhage patients low coated-platelet levels were associated with increased mortality at 1-month (76, 77). The association of low levels of coated-platelets with bleeding events was also noted in a study aimed at evaluating the diagnostic utility of platelet flow cytometry analysis in haemorrhagic diathesis patients with normal standard laboratory workup (80).

Interestingly, a recent study utilizing a microfabricated chip capable of measuring individual platelet contractile forces identified platelet subpopulations with varying contractility (81). This platelet-contraction cytometry revealed generally high levels of highly-contractile platelets in healthy donors. These were absent in patients with Wiskott-Aldrich syndrome, and a subpopulation of platelets with low-contractility was also noted among a subset of patients with chronic bleeding but normal clinical haemostasis tests. Whether subpopulations of high- and low-contractile platelets correspond to subpopulations of coated- or aggregating-platelet subpopulations remains to be studied.

Older studies characterizing platelets based on buoyant density and/or volume also demonstrated differences between platelets of healthy controls and patients. Using continuous gradients of Percoll to isolate platelets, it was shown that compared to healthy controls insulin-dependent diabetics with poor glycemic control have lower density platelets but with apparently normal granule levels (82). The change in density profiles was proposed to be due to abnormal platelet subpopulations, although what appeared to be log normally distributed populations were observed (83). A shift toward an increase in low-density platelet subpopulations was also observed in patients with hypercholesterolemia and it was argued that these platelets are more reactive in response to agonist stimulation (83). Conversely, a small increase in platelet size with increased surface exposure of integrin α_IIb_β_3_ was noted following acute myocardial infarction (84).

Interestingly, recent preliminary studies suggest that FACS separated platelets into the smallest and largest 10% may differ in some of their RNA transcripts (85). A sub-analysis of the RNA profiles of these platelet subpopulations demonstrated a trend in which the large-platelet subpopulation RNA profile was associated with hemostasis and wound healing, while the small-platelet subpopulation profile was associated with vascular cell function likely reflecting a potential differential ability to uptake RNA from vascular cells. The differential uptake of RNA by platelet subpopulations may allow for these subpopulations to serve as novel biomarkers for various diseases. In this context, tumor-educated platelets (platelets containing tumor-associated mRNA) have been identified as a potential platform for blood-based liquid biopsies for cancer (86, 87). How platelet subpopulations change in cancer and how these changes impact their mRNA content is unknown but likely of important significance to liquid biopsy utility.

## Differential Responses of Platelet Subpopulations to Anti-platelet Drugs

Less is still known about how various platelet subpopulations respond to anti-platelet drugs. Using counterflow centrifugation to isolate platelet subpopulations of different volume, Jakubowski et al. (88) demonstrated a correlation between increasing mean platelet volume and prostacyclin concentration necessary to inhibit aggregation suggesting greater platelet mass is associated with decreased inhibitory effect of prostacyclin. Similar, a study of FACS-sorted platelets into the 20% smallest and largest subpopulations showed that following incubation with acetylsalicylic acid (ASA) large platelets generate more thromboxane B_2_ (TXB_2_) compared to the small platelet subpopulation (89). However, the percent reduction in TXB_2_ generation caused by ASA tended to be greater in the small platelet subpopulation. Potential differences in response to anti-platelet therapy by platelet subpopulations may be clinically important as Hoefer et al. (90) have shown that subpopulations of drug-free/uninhibited platelets can either intermingle with ASA-inhibited platelets within an aggregate or form aggregate cores around which ASA- and P2Y_12_-inhibited platelets may activate potentially seeding thrombus formation.

## Challenges and Future Directions

Some of the challenges with studying platelet subpopulations are their identification, labeling and separating for functional studies. In the past these challenges were met with advances in research methodology and technology, such as the application of flow cytometry to platelet studies (91, 92). Recent advances in cytometry (Table 1) that have enhanced our ability to study platelet subpopulations include that application of fluorescence activated cell sorting and confocal microscopy (47), laser scanning cytometry (93), interfacial platelet cytometry (94), platelet-contraction cytometry (81), and mass cytometry (95). Moreover, the application of flowRNA technology to platelets studies may further help with RNA profiling of platelet subpopulations (96). These technologies will not only be crucial in aiding to delineate the roles of various platelet subpopulations in hemostasis and thrombosis, but also to understanding their potential differing roles in wound healing, angiogenesis, malignancy, and immunity and inflammation. Lastly, isolation of functionally distinct platelet subpopulations may be desirable for platelet concentrate preparation and various transfusion medicine applications (97, 98).

**Table 1:** List of cytometry techniques conducive to platelet subpopulation studies.

**Technique**	**Some major advantages for platelet subpopulation studies**	**References**
Confocal/epifluorescence microscopy	• Visualization of discrete subpopulations • Compatible with functional studies	(47, 56)
Flow cytometry (FC)	• Rapid, multi-parameter characterization of subpopulations	(47, 58)
Fluorescence activated cell sorting (FACS)	• Allows for separation of platelet subpopulations for further analysis	(47, 85)
Laser scanning cytometry (LSC)	• Rapid measurement of fluorescence and light scatter of slide-based specimens • Microscope-based enables visualization and morphological assessment	(93)
Interfacial platelet cytometry (iPC)	• Minimal sample preparation • Allows for studying subpopulation interactions with protein surfaces	(94)
Platelet contraction cytometry	• Enables measurement of contractile forces of individual platelets adhering on substrates	(81)
Mass cytometry	• Detects multiple heavy metal isotope-conjugated antibodies on platelet surface • Overcomes challenges with spectral overlap and eliminates need for compensation associated with FC • Greatly increases number of parameters that can be analyzed	(95)
FlowRNA	• Enables RNA measurement within specific cell subpopulations due to concurrent measurement of protein expression • Capacity to multiplex RNA measurement	(96)

## Author Contributions

GL and PJ contributed to manuscript generation, editing, and approval of the submitted manuscript.

### Conflict of Interest Statement

The authors declare that the research was conducted in the absence of any commercial or financial relationships that could be construed as a potential conflict of interest.
